# Peritumoral Adipose Tissue Features Derived from [^18^F]fluoro-2-deoxy-2-d-glucose Positron Emission Tomography/Computed Tomography as Predictors for Response to Neoadjuvant Chemotherapy in Breast Cancer Patients

**DOI:** 10.3390/jpm14090952

**Published:** 2024-09-09

**Authors:** Jeong Won Lee, Yong Kyun Won, Hyein Ahn, Jong Eun Lee, Sun Wook Han, Sung Yong Kim, In Young Jo, Sang Mi Lee

**Affiliations:** 1 Department of Nuclear Medicine, Soonchunhyang University Cheonan Hospital, 31 Suncheonhyang 6-gil, Dongnam-gu, Cheonan 31151, Republic of Korea; 2 Department of Radiation Oncology, Soonchunhyang University Cheonan Hospital, 31 Suncheonhyang 6-gil, Dongnam-gu, Cheonan 31151, Republic of Korea; 3 Department of Pathology, CHA Gangnam Medical Center, CHA University School of Medicine, 569 Nonhyon-ro, Gangnam-gu, Seoul 06135, Republic of Korea; 4 Department of Surgery, Soonchunhyang University Cheonan Hospital, 31 Suncheonhyang 6-gil, Dongnam-gu, Cheonan 31151, Republic of Korea

**Keywords:** adipose tissue, breast cancer, neoadjuvant chemotherapy, positron emission tomography, texture analysis

## Abstract

This study investigated whether the textural features of peritumoral adipose tissue (AT) on [^18^F]fluoro-2-deoxy-2-d-glucose (FDG) positron emission tomography/computed tomography (PET/CT) can predict the pathological response to neoadjuvant chemotherapy (NAC) and progression-free survival (PFS) in breast cancer patients. We retrospectively enrolled 147 female breast cancer patients who underwent staging FDG PET/CT and completed NAC and underwent curative surgery. We extracted 10 first-order features, 6 gray-level co-occurrence matrix (GLCM) features, and 3 neighborhood gray-level difference matrix (NGLDM) features of peritumoral AT and evaluated the predictive value of those imaging features for pathological complete response (pCR) and PFS. The results of our study demonstrated that GLCM homogeneity showed the highest predictability for pCR among the peritumoral AT imaging features in the receiver operating characteristic curve analysis. In multivariate logistic regression analysis, the mean standardized uptake value (SUV), 50th percentile SUV, 75th percentile SUV, SUV histogram entropy, GLCM entropy, and GLCM homogeneity of the peritumoral AT were independent predictors for pCR. In multivariate survival analysis, SUV histogram entropy and GLCM correlation of peritumoral AT were independent predictors of PFS. Textural features of peritumoral AT on FDG PET/CT could be potential imaging biomarkers for predicting the response to NAC and disease progression in breast cancer patients.

## 1. Introduction

Neoadjuvant chemotherapy (NAC) is a standard treatment option for locally advanced breast cancer, especially preferred for human epidermal growth factor receptor 2 (HER2)-enriched or triple-negative breast cancer with a tumor size > 2 cm and/or axillary lymph node metastasis [1]. Compared to conventional adjuvant chemotherapy, NAC does not significantly improve survival outcomes [2]. However, NAC can reduce the size and extent of breast cancer lesions, increasing the likelihood of tumor control and enabling more patients to undergo conservative breast surgery [3,4]. Furthermore, NAC also provides an opportunity for the early assessment of chemotherapy efficacy [5]. Pathological complete response after NAC is associated with better survival in several meta-analysis studies and has been considered as a surrogate endpoint marker for predicting prognosis in patients with breast cancer [3,6,7]. Therefore, several studies have attempted to predict pathological responses before the completion of NAC or surgery using imaging examinations [8,9]. One of the imaging modalities used in previous studies is [^18^F]fluoro-2-deoxy-2-d-glucose (FDG) positron emission tomography/computed tomography (PET/CT) which is useful in detecting metastasis and predicting prognosis in patients with breast cancer [8,10,11]. In addition to conventional PET/CT parameters such as the maximum standardized uptake value (SUV), metabolic tumor volume (MTV), and total lesion glycolysis (TLG), the radiomic features of PET images extracted from textural analysis, which can quantify the degree of intratumoral metabolic heterogeneity, have recently been used to predict responses to NAC [12,13]. However, the accuracy of PET/CT features of primary breast cancer lesions in predicting pathological response to NAC is only moderate revealing inconsistent results between previous studies [8,14].

Because the mammary gland is surrounded mainly by mammary adipose tissue (AT), AT is a major component of the tumor microenvironment of breast cancer cells [15]. Recently, breast cancer cells have been found to interact with diverse cells in peritumoral AT, such as adipocytes and AT-derived mesenchymal stromal/stem cells, which contribute profoundly to the proliferation, invasion, metastasis, and resistance to therapy of breast cancer cells [15,16,17]. Due to increased glycolysis of adipocytes and recruitment of immune cells in the peritumoral AT, these interactions lead to metabolic alterations in the peritumoral AT [17,18]; therefore, several studies have demonstrated that imaging features of peritumoral AT on FDG PET/CT could reflect metabolic changes in the peritumoral AT and have a clinical significance [19,20,21]. Considering that cross-talk between breast cancer cells and peritumoral AT cells facilitates tumor progression and chemotherapy resistance [15,16], the textural features of peritumoral AT on FDG PET/CT could have a significant relationship with response to NAC. However, the predictive value of peritumoral AT textural features on FDG PET/CT in patients with breast cancer has not been reported yet.

Therefore, this study aimed to investigate the clinical value of peritumoral AT textural features on FDG PET/CT in predicting the response to NAC and progression-free survival (PFS) in patients with breast cancer.

## 2. Materials and Methods

### 2.1. Patients

We retrospectively reviewed the medical records of female patients with invasive breast cancer at two medical centers (International St. Mary’s Hospital and Soonchunhyang University Cheonan Hospital) between January 2013 and December 2020. The patients (1) who underwent staging FDG PET/CT prior to NAC, (2) who received NAC and subsequent surgical resection of breast cancer, and (3) whose imaging, histopathology, and follow-up data were available were included in the study. The exclusion criteria were as follows: (1) distant metastasis (stage M1) on staging work-up examinations, (2) no surgery after NAC, (3) insufficient peritumoral breast AT volume for extraction of textural features, (4) diffuse infiltrative type of breast cancer on imaging studies (diffuse, infiltrative, and non-mass enhancement on magnetic resonance imaging [MRI] and diffusely increased FDG uptake in PET/CT), (5) loss to follow-up within 1 year after the surgery, and (6) history of previous malignant diseases. Based on the inclusion and exclusion criteria, 147 patients were included in this study.

### 2.2. Treatment and Response Assessment

All the patients underwent blood tests, breast ultrasonography, bone scintigraphy, contrast-enhanced chest CT, breast MRI, and FDG PET/CT for a staging workup. After staging examinations, the patients were treated with one of the following five NAC regimens: (1) doxorubicin and cyclophosphamide; (2) doxorubicin and docetaxel; (3) doxorubicin, cyclophosphamide, and docetaxel; (4) docetaxel, carboplatin, trastuzumab, and pertuzumab; and (5) doxorubicin, cyclophosphamide, paclitaxel, and trastuzumab. Following NAC, mastectomy or breast-conserving surgery with sentinel lymph node biopsy and/or axillary lymph node dissection was performed. The pathological tumor response to NAC was determined by histopathological evaluation of the surgical specimens. Patients with the complete absence of both residual invasive cancer cells and cancer in situ cells in the breast and lymph nodes (ypT0N0), were classified as having a pathological complete response (pathological complete responders). Other patients who showed a partial response, stable disease, or disease progression after NAC were classified as non-responders. All patients received adjuvant chemotherapy, radiotherapy, and/or hormonal treatment after surgery. After treatment, regular clinical follow-ups were conducted every 3–6 months with blood and imaging examinations including breast ultrasonography and contrast-enhanced chest CT.

### 2.3. FDG PET/CT and Textural Analysis

FDG PET/CT was performed using a Biograph mCT 20 scanner (Siemens Healthineers, Knoxville, TN, USA) at International St. Mary’s Hospital and a Biograph mCT 128 scanner (Siemens Healthineers) at Soonchunhyang University Cheonan Hospital. All the patients were required to fast for at least 6 h before FDG injection. At the time of FDG injection, blood glucose levels were below 150 mg/dL. FDG was injected intravenously 60 ± 3 min before PET/CT scan at a dose of approximately 4.07 MBq/kg for both scanners. All PET/CT scans were performed from the skull base to the proximal thigh in the supine position. Initially, a non-contrast-enhanced CT scan was performed for attenuation correction at 80 mA and 100 kVp for the Biograph mCT 20 scanner and at 100 mA and 120 kVp for the Biograph mCT 128 scanner. All CT scans were performed using an automated dose modulation with a slice thickness of 5 mm. Subsequently, a PET scan was performed for 1.5 min per bed position in both PET/CT scanners. For both scanners, PET images were reconstructed using the ordered-subset expectation maximization algorithm with the point spread function, time-of-flight modeling, and attenuation correction on a 128 × 128 matrix (2 iterations and 21 subsets, voxel size of 4.0 × 4.0 × 3.0 mm^3^).

**Figure 1:**
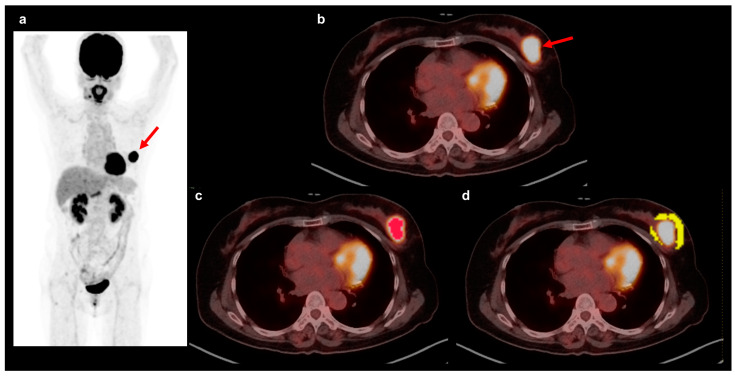
Imaging parameters of the primary tumor and textural features of peritumoral AT. Maximum intensity projection image (**a**) and transaxial fused PET/CT images (**b**–**d**) of FDG PET/CT are shown to illustrate an example of VOI for measuring imaging parameters of primary breast cancer lesions and textural features of peritumoral AT. A 63-year-old woman underwent FDG PET/CT for staging of left breast cancer. The breast cancer lesion was histopathologically confirmed as invasive ductal carcinoma, HER2-enriched subtype, demonstrating intensely increased FDG uptake on PET/CT images with a maximum SUV of 19.2 (arrows on (**a**,**b**)). For the primary breast cancer lesion, a VOI was drawn manually around the breast cancer lesion, and an area with an SUV higher than 40% of the maximum SUV was automatically selected within the VOI (area in red in (**c**)). For peritumoral AT, a VOI that covers the area within 1 cm from the margin of the primary breast cancer was drawn manually, and the areas that had CT attenuation ranging between −200 and −50 HU were automatically selected within the VOI (area in yellow in (**d**)).

Two nuclear medicine physicians who were blinded to the patient’s clinical, pathological, and survival data measured the quantitative imaging parameters of the primary tumor and performed textural analysis of the peritumoral AT on FDG PET/CT through consensus (Figure 1). The open-source software LIFEx software version 7.0.0 (www.lifexsoft.org) was used for PET/CT imaging analysis [22]. A volume of interest (VOI) was manually drawn around the primary breast cancer lesion and the maximum standardized uptake value (SUV) of the breast cancer was measured. Using a threshold SUV of 40% of the maximum SUV, voxels with an SUV greater than the threshold SUV were automatically defined within the VOI, and the MTV and mean SUV of the voxels were measured. Furthermore, TLG was calculated as MTV × mean SUV. The peritumoral AT area was segmented using a previously described method [19]. The VOI was drawn manually to include the primary breast cancer and surrounding breast tissue within a 1.0 cm distance from the tumor margin. Within the VOI, areas of the surrounding breast tissue with CT attenuation ranging between −200 and −50 Hounsfield unit (HU) were automatically selected and defined as the peritumoral AT. Prior to extracting textural features, spillover FDG activity of the breast cancer lesion in the peritumoral AT was removed manually. From the peritumoral AT, 19 textural features of PET images were extracted, which comprised 10 first-order features, 6 gray-level co-occurrence matrix (GLCM) features, and 3 neighborhood gray-level difference matrix (NGLDM) features (Table S1). The 10 first-order features consisted of the maximum, mean, standard deviation, 25th percentile, 50th percentile, and 75th percentile values of the SUV and kurtosis, skewness, energy, and entropy calculated from the SUV histogram. The six GLCM features were contrast, correlation, dissimilarity, energy, entropy, and homogeneity, whereas the three NGLDM features were busyness, coarseness, and contrast [23,24]. For computing textural features of the peritumoral AT, PET images were reconstructed into a voxel size of 4.07 × 4.07 × 2.5 mm^3^. Prior to extracting textural features from grey-level analyses, intensity levels of FDG uptake of voxels were resampled into 64 relative gray levels. GLCM features were computed using a distance of one voxel in 13 directions, and NGLDM features were computed in three directions with 26 neighbor voxels.

### 2.4. Statistical Analysis

**Figure 2:**
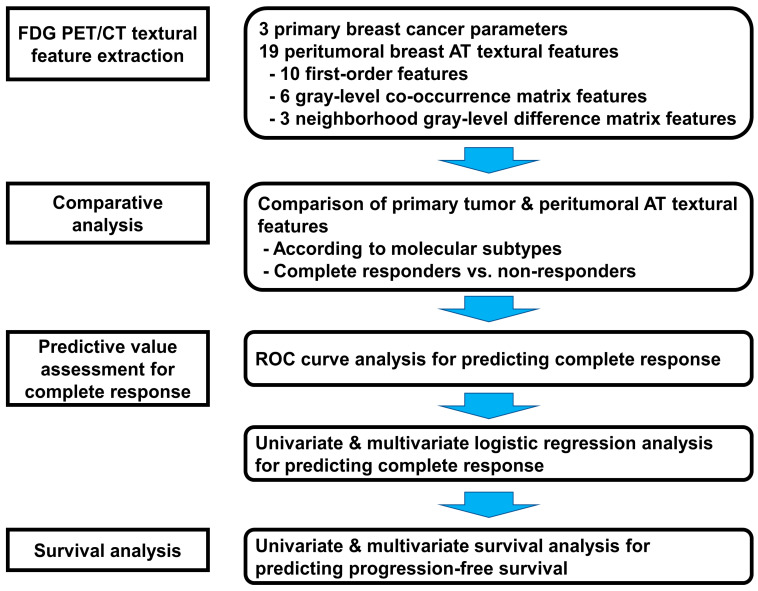
Schematic overview of the workflow of the present study.

A schematic presentation of the workflow in the current study is depicted in Figure 2. The Shapiro–Wilk test was used to assess the normality of the distribution. Because all FDG PET/CT parameters of the primary breast cancer and peritumoral AT were abnormally distributed, they were described as medians with interquartile ranges. The Kruskal–Wallis test with post hoc Dunn’s test and the Mann–Whitney test were conducted to evaluate differences in imaging parameters of the primary breast cancer and textural features of the peritumoral AT according to molecular breast cancer subtypes and pathological response, respectively. The receiver operating characteristic (ROC) curve analysis was performed to calculate the area under the ROC curve (AUC) value and evaluated the ability of PET/CT imaging features to predict pathological complete response. The optimal cut-off values of the PET/CT parameters were determined using the Youden index. The sensitivity and specificity of the parameters for predicting pathological complete response were identified using the optimal cut-off values. Univariate and multivariate logistic regression analyses were conducted to assess independent predictors of pathological response among the PET/CT parameters. PFS was defined as the period between the day of NAC initiation and the day of detection of disease progression, death, or the last clinical follow-up. Univariate and multivariate Cox proportional hazard regression analyses were performed to evaluate the prognostic value of PET/CT parameters for predicting PFS. For both logistic regression and survival analyses, only statistically significant PET/CT parameters in the univariate analysis were included in the multivariate analysis. Age, clinical TNM stage, and molecular subtype were added as covariates in the multivariate analysis. Considering the number of events, each PET/CT parameter was assessed in a separate model. To estimate the survival curves, the optimal cut-off values for the PET/CT parameters were selected using ROC curve analysis. According to the cut-off values, PFS curves were calculated using Kaplan–Meier analysis with a log-rank test. All statistical analyses were conducted using MedCalc Statistical Software version 20.218 (MedCalc Software Ltd., Ostend, Belgium). The level of statistical significance was set at *p* < 0.05.

## 3. Results

### 3.1. Patient Characteristics and Pathological Outcomes

Baseline characteristics of the 147 enrolled female patients with invasive breast cancer are summarized in Table 1. Of the 147 patients, 141 (95.9%) were diagnosed with invasive ductal carcinoma and 2 patients (1.4%) were diagnosed with invasive lobular carcinoma, mucinous carcinoma, and papillary carcinoma. Among the patients, 136 (92.5%) showed lymph node metastasis on pretreatment examinations and 107 (72.8%) had clinical TNM stage III. In the evaluation of the pathological tumor response to NAC, 36 patients (24.5%) and 111 patients (75.5%) were classified into the pathological complete responder and non-responder groups, respectively.

### 3.2. PET/CT Textural Features and Molecular Subtypes

Results of the comparative analysis of the imaging parameters of primary breast cancer and textural features of peritumoral AT according to the molecular subtypes of breast cancer are shown in Table 2. Among the primary breast cancer imaging parameters, there were significant differences in maximum SUV and TLG according to the molecular subtypes. Among the textural features of peritumoral AT, NGLDM coarseness was significantly correlated with molecular subtypes (*p* < 0.05). Post hoc analyses showed that HER-enriched and triple-negative breast cancers had significantly higher maximum SUV values than all other breast cancer types and higher TLG than luminal A breast cancer (*p* < 0.05). Both subtypes had significantly lower NGLDM coarseness values than the luminal A type (*p* < 0.05). Furthermore, SUV histogram kurtosis, SUV histogram entropy, GLCM entropy, and NGLDM busyness of peritumoral AT were correlated with molecular subtypes; the statistical significance was borderline (*p* < 0.10).

### 3.3. PET/CT Textural Features and Pathological Response

Imaging parameters of primary breast cancer and textural features of peritumoral AT were compared between pathological complete responders and non-responders (Table 3). Among the textural features of peritumoral AT, responders showed significantly lower values of the mean, 25th percentile, 50th percentile, and 75th percentile values of SUV, SUV histogram entropy, GLCM dissimilarity, and GLCM entropy, and significantly higher values of GLCM homogeneity than non-responders (*p* < 0.05). In contrast, there was no significant difference in imaging parameters of primary breast cancer between both groups (*p* > 0.05).

**Figure 3:**
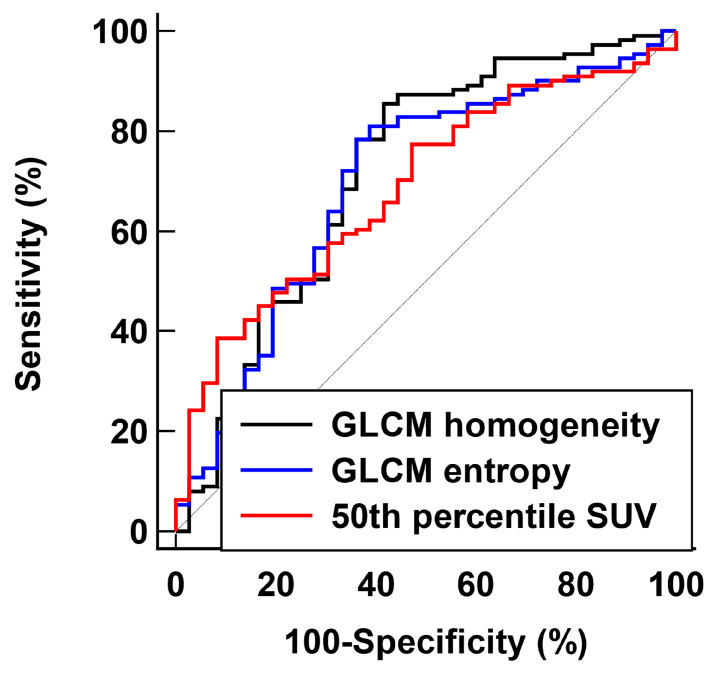
ROC curves analysis of PET/CT parameters to identify predictors of pathological complete response.

In the ROC curve analysis, GLCM homogeneity revealed the highest predictability for identifying pathological complete response among PET/CT parameters, with an AUC of 0.717 (95% confidence interval [CI], 0.600–0.811), followed by GLCM entropy (AUC, 0.697; 95% CI, 0.585–0.791) and 50th percentile SUV (AUC, 0.686; 95% CI, 0.583–0.768; Table 4; Figure 3). Using an optimal cut-off value of 0.75, GLCM homogeneity showed a sensitivity of 58.3% and a specificity of 85.6% for predicting pathological complete response, whereas 50th percentile SUV showed a sensitivity of 91.7% and a specificity of 38.7% using a cut-off value of 0.78.

The relationship between pathological complete response and FDG PET/CT imaging features was further analyzed using univariate and multivariate logistic regression analyses (Table 5). Mean, 25th percentile, 50th percentile, and 75th percentile values of SUV, SUV histogram entropy, GLCM energy, GLCM entropy, and GLCM homogeneity of the peritumoral AT were significantly associated with pathological complete response in the univariate analysis (*p* < 0.05). Significant imaging features in the univariate analysis were included in the multivariate analysis, adding age, clinical TNM stage, and molecular subtype as covariates. In the multivariate analysis, mean SUV (*p* = 0.012), 50th percentile SUV (*p* = 0.002), 75th percentile SUV (*p* = 0.015), SUV histogram entropy (*p* = 0.034), GLCM entropy (*p* = 0.001), and GLCM homogeneity (*p* = 0.002) were independent predictors for pathological complete response. Higher values of GLCM homogeneity and lower values of the mean, 25th percentile, and 75th percentile values of SUV, SUV histogram entropy, and GLCM entropy were associated with higher pathological complete response rates.

### 3.4. Survival Analysis for PFS

**Figure 4:**
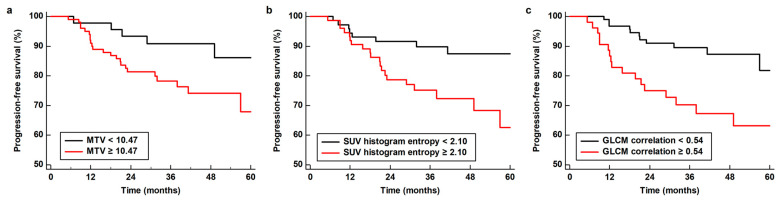
Factors for predicting PFS. Kaplan–Meier analysis of PFS according to MTV of primary breast cancer (**a**) and SUV histogram entropy (**b**) and GLCM correlation (**c**) of peritumoral AT.

The patients’ median PFS was 36.9 months (range, 5.6–90.5 months). During the follow-up, disease progression or death was observed in 29 patients (19.7%). The association between FDG PET/CT features and PFS was assessed using a Cox proportional hazards regression model (Table 6). In the univariate analysis, along with MTV and TLG of the primary breast cancer lesion, 50th percentile value of SUV, SUV histogram entropy, GLCM correlation, GLCM entropy, and NGLDM coarseness of the peritumoral AT were significantly associated with PFS (*p* < 0.05). Significant PET/CT imaging features were included in the multivariate survival analysis after adjusting for age, clinical TNM stage, and molecular subtype. In the multivariate analysis, MTV of the primary breast tumor (*p* = 0.039; hazard ratio, 1.01 per 1.00 cm^3^ increase; 95% CI, 1.00–1.02) and SUV histogram entropy (*p* = 0.042; hazard ratio, 2.71 per 1.00 increase; 95% CI, 1.10–10.54) and GLCM correlation (*p* = 0.040; hazard ratio, 29.32 per 1.00 increase; 95% CI, 1.17–734.56) of the peritumoral AT were independent predictors for PFS. An increased MTV of the primary tumor, increased SUV histogram entropy and GLCM correlation of peritumoral AT were associated with poor survival. When the Kaplan–Meier survival curves were generated, patients with high values of MTV (≥10.47 cm^3^), SUV histogram entropy (≥2.10), and GLCM correlation (≥0.54) had a significantly worse PFS than those with low values (*p* = 0.035, *p* = 0.007, and *p* = 0.001, respectively; Figure 4).

## 4. Discussion

Nowadays, there is ample evidence that breast cancer cells have intimate bidirectional communication with peritumoral AT cells via cell-to-cell interactions and secretion of bioactive factors, including cytokines and chemokines [15]. Breast cancer cells modify adipocytes and AT-derived mesenchymal stromal/stem cells into cancer-associated adipocytes and cancer-associated fibroblasts with decreased differentiation marker expression and increased expression of pro-tumoral and pro-inflammatory molecules [15,16,17]. In previous studies, modifications of AT cells were more prominently found in peritumoral AT than in AT distant from the tumors [16,25]. These modified cells play a crucial role in promoting growth, metastasis, angiogenesis, and therapy resistance of breast cancer tissue and recruiting immunosuppressive cells in the peritumoral AT [15,16,26]. 

The cross-talk between cancer cells and peritumoral AT cells can alter FDG uptake of peritumoral AT through two mechanisms. One is the Warburg effect induced by cancer cells in cancer-associated adipocytes [18]. In a previous study, breast cancer cells induced overexpression of 5′-adenosine monophosphate-activated protein kinase (AMPK) and hexokinase 2 (HK2) in cancer-associated adipocytes located in the peritumoral AT [18]. As both AMPK and HK2 are the main enhancers of glucose uptake and glycolysis in cells, this phenomenon leads to increased FDG uptake in cancer-associated adipocytes [18]. Another mechanism is increased inflammatory changes and recruitment of immune cells in peritumoral AT [15,27]. By secreting diverse pro-inflammatory cytokines and chemokines, cancer-associated adipocytes recruit immunosuppressive cells such as tumor-associated macrophages and regulatory T-cells and evoke an inflammatory response in peritumoral AT [15,27,28]. Of immune cells, M2-subtype macrophages, which are associated with tumor progression and immune suppression in the tumor microenvironment, are associated with FDG uptake in peritumoral AT [19,29]. In previous immunohistochemical analyses of surgical specimens of gastric and colorectal cancers, increased M2-subtype macrophage infiltration in peritumoral AT was significantly related to increased mean SUV and metabolic heterogeneity of peritumoral AT on FDG PET/CT [20,21]. Considering the significant relationship between FDG PET/CT findings and cancer-induced modifications in peritumoral AT, it is not surprising that FDG PET/CT textural features of peritumoral AT could have clinical implications in predicting tumor aggressiveness and prognosis. In previous studies, the textural features of peritumoral AT on FDG PET/CT turned out to be independent predictors of recurrence-free survival after curative surgery in patients with gastric and colorectal cancers [20,21]. In patients with breast cancer, a previous study extracted 38 PET/CT textural features of peritumoral and contralateral AT from the data of 326 patients with breast cancer [19]. In that study, 37 textural features showed significant differences between peritumoral and contralateral AT, indicating increased FDG uptake and metabolic heterogeneity in peritumoral AT. Moreover, the textural features of peritumoral AT demonstrated a high diagnostic ability for predicting axillary lymph node metastasis of breast cancer, which was comparable to that of maximum SUV of lymph node. Therefore, peritumoral AT showing increased FDG uptake and metabolic heterogeneity on PET/CT can be considered to have a more robust interrelationship with cancer cells, which further increases the risk of cancer metastasis and recurrence.

In this study, we investigated whether the textural features of peritumoral AT on FDG PET/CT have clinical value in predicting the pathological response to NAC in patients with breast cancer. We found that the mean, 50th percentile, and 75th percentile values of SUV, SUV histogram entropy, GLCM entropy, and GLCM homogeneity of peritumoral AT were independent predictors of pathological complete response to NAC, with higher values of the mean, 50th percentile, and 75th percentile values of SUV, SUV histogram entropy, and GLCM entropy and a lower value of GLCM homogeneity in non-responders than responders. The mean, 50th percentile, and 75th percentile values of SUV represent the degree of FDG uptake. SUV histogram entropy and GLCM entropy measure the randomness of the SUV intensity distribution within an image; a high entropy value indicates an image with a random distribution of SUV intensities [21,30]. GLCM homogeneity measures the uniformity of pixel pairs, and a high GLCM homogeneity value indicates many voxels with similar SUV intensities in an image [31]. Hence, the results of our study implied that patients with breast cancer who showed increased FDG uptake intensity and metabolic heterogeneity in the peritumoral AT were less likely to have a chance of pathological complete response to NAC, suggesting the role of PET/CT textural features of peritumoral AT as potential imaging biomarkers for predicting the response to NAC. The results of this study provide imaging evidence that interactions between breast cancer cells and peritumoral AT cells are involved chemotherapy resistance. Recently, to improve breast cancer treatment effects, cancer-associated adipocytes, and cancer-associated fibroblasts have been targeted [15,32]. Drugs that target these cells themselves or bioactive molecules secreted from the cells have been shown to reduce breast cancer cell growth and resistance to chemotherapy [15,32]. In future clinical studies targeting cancer-associated AT, the textural features of peritumoral AT might help in selecting optimal candidates.

In our study, two textural features of peritumoral AT, SUV histogram entropy and GLCM correlation, were significantly associated with PFS in the multivariate survival analysis, along with the MTV of the primary tumor. The GLCM correlation measures the linear dependence of SUV intensity in an image [31]. It evaluates whether there is a linear relationship of the SUV intensity between the two neighboring pixels in an image, and, in previous studies, patients with malignant tumors with high GLCM correlation values had significantly poor survival [33]. Although both SUV histogram entropy and GLCM correlation were independent predictors of PFS, SUV histogram entropy might be more suitable for use as an imaging biomarker because it also showed a significant association with NAC response in our study and the presence of axillary lymph node metastasis in a previous study [19]. Furthermore, textural features based on SUV histogram reflect the global heterogeneity of a lesion and are known to be more stable and reliable than GLCM features [8]. However, because textural features of peritumoral AT only represent cancer-associated changes in the peritumoral AT, incorporating both tumor and peritumoral AT imaging features could improve the predictability of clinical outcomes in patients with breast cancer [30].

In the comparative analyses of FDG PET/CT imaging features according to molecular subtypes of breast cancer, HER-enriched and triple-negative breast cancers showed significantly higher maximum SUV values than all other breast cancer types, as shown in previous studies [34]. Furthermore, among the peritumoral AT imaging features, HER-enriched and triple-negative breast cancers showed significantly lower NGLDM coarseness values than the luminal A type, and several textural features including SUV histogram kurtosis, SUV histogram entropy, GLCM entropy, and NGLDM busyness showed borderline significance. NGLDM coarseness measures the average difference between the central voxel and its neighborhood voxels, and a high value of NGLDM coarseness indicates a more uniform texture in an image [35]. Considering that HER-enriched and triple-negative breast cancers show different characteristics in the tumor microenvironment than other subtypes [36], it seems that these differences in the tumor microenvironment also affected the peritumoral AT imaging features on FDG PET/CT. 

This study had several limitations. The retrospective design of this study is the first limitation, which might contain a certain risk of bias. Further, the results of the current study should be externally validated with larger patient populations. Second, textural analysis has been widely used to quantify imaging features; however, the lack of standardization of analytical methods is still a major hurdle to the general clinical applications of textural features [37]. Third, since molecular subtypes of breast cancer, NAC regimens, and types of adjuvant treatment modalities can affect the response to NAC and survival of breast cancer patients, further evaluation of the clinical significance of peritumoral AT imaging parameters according to those factors might be needed. Fourth, although AT within a 1 cm distance of the tumor margin has been applied to define peritumoral AT on FDG PET/CT in previous studies, the definition of peritumoral AT for extracting textural features should be further validated [19,20,21]. Further studies are necessary to establish the method for measuring textural features of peritumoral AT. Finally, there might be a mild limitation arising from analyzing images from two different PET/CT scanners, even though the two scanners were from the same company (Siemens Healthineers) and adopted the same acquisition protocol for PET images.

## 5. Conclusions

The present study demonstrated that the textural features of peritumoral AT on FDG PET/CT could predict the response to NAC and PFS in patients with breast cancer. Increased FDG uptake intensity and metabolic heterogeneity of peritumoral AT were associated with poor response to NAC and worse PFS. The textural features of peritumoral AT on FDG PET/CT could be potential imaging biomarkers for predicting clinical outcomes in patients with breast cancer treated with NAC.

## Figures and Tables

**Table 1:** Patients’ clinicopathological characteristics.

Characteristics	Number of Patients (Percentage) *n*= 147
Median age, years (range)	47 (23–79)
Obesity	
Underweight/normal	56 (38.1%)
Overweight/obesity	91 (61.9%)
Menopausal status	
Premenopausal	80 (54.4%)
Postmenopausal	67 (45.6%)
Histopathology	
Invasive ductal carcinoma	141 (95.9%)
Others	6 (4.1%)
Histologic grade	
Grade 1	15 (10.2%)
Grade 2	79 (53.7%)
Grade 3	49 (33.3%)
Not specified	4 (2.7%)
Molecular subtypes	
Luminal A	28 (19.0%)
Luminal B-like HER2-negative	19 (12.9%)
Luminal B-like HER2-positive	60 (40.8%)
HER2-enriched	21 (14.3%)
Triple-negative	19 (12.9%)
Clinical T stage	
T1–T2	90 (61.2%)
T3–T4	57 (38.8%)
Clinical N stage	
N0	11 (7.5%)
N1	63 (42.9%)
N2–N3	73 (49.7%)
Clinical TNM stage	
Stage II	40 (27.2%)
Stage III	107 (72.8%)
Neoadjuvant chemotherapy regimen	
Doxorubicin and docetaxel	52 (35.4%)
Doxorubicin, cyclophosphamide, and docetaxel	40 (27.2%)
Docetaxel, carboplatin, trastuzumab, and pertuzumab	25 (17.0%)
Doxorubicin and cyclophosphamide	22 (15.0%)
Doxorubicin, cyclophosphamide, paclitaxel, and trastuzumab	8 (5.4%)

HER2, human epidermal growth factor receptor 2.

**Table 2:** Primary breast cancer imaging parameters and textural features of peritumoral AT according to molecular subtypes.

Parameters	Luminal A	Luminal B-like HER2 Negative	Luminal B-like HER2 Positive	HER2-Enriched	Triple Negative	*p*-Value
Primary tumor						
Maximum SUV	7.79 (5.58–11.66)	8.17 (6.26–9.56)	10.04 (5.19–14.75)	14.02 (10.82–18.36)	13.01 (9.51–18.13)	<0.001
MTV	6.76 (3.61–10.95)	10.47 (5.25–21.84)	8.45 (3.87–18.85)	9.06 (5.39–14.63)	8.79 (4.61–13.72)	0.633
TLG	27.64 (19.13–75.96)	68.48 (20.07–93.17)	39.46 (20.67–82.19)	75.82 (52.18–144.57)	79.93 (31.42–134.41)	0.039
Peritumoral AT						
First-order features						
Maximum SUV	2.44 (2.22–2.80)	2.43 (2.26–2.58)	2.53 (2.10–2.86)	2.47 (2.23–2.83)	2.52 (2.36–2.96)	0.440
Mean SUV	0.74 (0.66–0.89)	0.78 (0.64–0.86)	0.80 (0.69–0.90)	0.78 (0.69–0.87)	0.79 (0.70–0.97)	0.770
Standard deviation SUV	0.35 (0.34–0.39)	0.38 (0.34–0.43)	0.37 (0.30–0.43)	0.35 (0.30–0.45)	0.37 (0.34–0.48)	0.366
25th percentile SUV	0.46 (0.43–0.58)	0.46 (0.38–0.58)	0.52 (0.43–0.62)	0.54 (0.44–0.63)	0.49 (0.44–0.70)	0.473
50th percentile SUV	0.66 (0.57–0.79)	0.62 (0.58–0.75)	0.70 (0.60–0.80)	0.71 (0.60–0.78)	0.81 (0.60–0.89)	0.368
75th percentile SUV	0.93 (0.82–1.09)	0.99 (0.78–1.09)	0.97 (0.83–1.12)	0.92 (0.82–1.07)	0.95 (0.83–1.23)	0.927
SUV histogram kurtosis	4.32 (3.84–5.74)	4.10 (3.61–4.65)	4.93 (4.06–5.94)	5.27 (4.40–6.26)	5.59 (3.92–6.84)	0.071
SUV histogram skewness	1.14 (0.92–1.30)	1.05 (0.91–1.18)	1.21 (0.96–1.47)	1.27 (0.94–1.47)	1.37 (0.98–1.67)	0.294
SUV histogram energy	0.29 (0.25–0.31)	0.27 (0.25–0.30)	0.29 (0.26–0.34)	0.29 (0.25–0.35)	0.28 (0.22–0.32)	0.629
SUV histogram entropy	2.08 (2.03–2.22)	2.17 (2.05–2.31)	2.06 (1.88–2.25)	2.18 (1.84–2.33)	2.18 (2.09–2.60)	0.078
GLCM features						
Contrast	1.15 (0.94–1.65)	1.25 (0.88–1.79)	1.17 (0.85–1.56)	1.22 (0.97–1.43)	1.33 (0.96–2.00)	0.808
Correlation	0.50 (0.42–0.57)	0.51 (0.45–0.56)	0.48 (0.35–0.57)	0.47 (0.37–0.58)	0.57 (0.43–0.63)	0.519
Dissimilarity	0.70 (0.63–0.89)	0.73 (0.57–0.94)	0.71 (0.57–0.81)	0.72 (0.60–0.82)	0.74 (0.57–0.98)	0.837
Energy	0.14 (0.11–0.16)	0.13 (0.09–0.16)	0.15 (0.12–0.18)	0.13 (0.11–0.17)	0.13 (0.09–0.19)	0.421
Entropy	3.55 (3.16–3.81)	3.59 (3.40–4.10)	3.34 (3.03–3.82)	3.61 (3.15–3.89)	3.94 (3.41–4.13)	0.057
Homogeneity	0.72 (0.67–0.75)	0.70 (0.65–0.73)	0.72 (0.69–0.76)	0.71 (0.67–0.75)	0.70 (0.63–0.76)	0.554
NGLDM features						
Busyness	2.09 (1.39–3.89)	3.29 (2.72–4.81)	2.80 (1.68–4.12)	3.36 (2.44–4.29)	2.17 (1.48–3.00)	0.061
Coarseness	0.019 (0.011–0.039)	0.015 (0.009–0.019)	0.016 (0.009–0.029)	0.011 (0.008–0.014)	0.012 (0.009–0.015)	0.032
Contrast	0.028 (0.020–0.035)	0.027 (0.023–0.046)	0.027 (0.020–0.034)	0.023 (0.017–0.029)	0.029 (0.022–0.039)	0.215

All data are expressed as median (interquartile range). The *p*-values are the results of the Kruskal–Wallis test. AT, adipose tissue; GLCM, gray-level co-occurrence matrix; HER2, Human epidermal growth factor receptor 2; MTV, metabolic tumor volume; NGLDM, neighborhood gray-level difference matrix; SUV, standardized uptake value; TLG, total lesion glycolysis.

**Table 3:** Primary breast cancer imaging parameters and textural features of peritumoral AT according to responses.

Parameter	Responders (*n* = 36)	Non-Responders (*n* = 111)	*p*-Value
Primary tumor			
Maximum SUV	11.89 (7.57–15.68)	9.49 (6.25–14.81)	0.151
MTV	6.56 (3.29–15.51)	8.81 (4.29–15.21)	0.269
TLG	51.86 (21.58–123.98)	59.57 (20.83–91.38)	0.650
Peritumoral AT			
First-order features			
Maximum SUV	2.40 (2.21–2.76)	2.52 (2.24–2.82)	0.270
Mean SUV	0.72 (0.64–0.82)	0.80 (0.70–0.90)	0.011
Standard deviation SUV	0.35 (0.31–0.40)	0.37 (0.33–0.45)	0.137
25th percentile SUV	0.44 (0.40–0.56)	0.52 (0.45–0.64)	0.017
50th percentile SUV	0.60 (0.54–0.71)	0.71 (0.61–0.85)	<0.001
75th percentile SUV	0.89 (0.78–1.00)	0.99 (0.84–1.14)	0.009
SUV histogram kurtosis	5.05 (4.16–6.95)	4.68 (3.81–5.83)	0.121
SUV histogram skewness	1.31 (1.05–1.56)	1.17 (0.91–1.42)	0.072
SUV histogram energy	0.30 (0.27–0.34)	0.28 (0.25–0.32)	0.051
SUV histogram entropy	2.02 (1.88–2.18)	2.14 (2.01–2.35)	0.020
GLCM features			
Contrast	1.07 (0.83–1.36)	1.28 (0.96–1.71)	0.052
Correlation	0.49 (0.43–0.57)	0.50 (0.38–0.58)	0.986
Dissimilarity	0.66 (0.54–0.77)	0.74 (0.61–0.88)	0.028
Energy	0.14 (0.12–0.19)	0.13 (0.10–0.17)	0.054
Entropy	3.12 (2.94–3.65)	3.63 (3.32–3.94)	<0.001
Homogeneity	0.75 (0.70–0.78)	0.70 (0.66–0.74)	0.003
NGLDM features			
Busyness	3.10 (1.77–4.20)	2.80 (1.72–4.07)	0.604
Coarseness	0.015 (0.011–0.029)	0.014 (0.009–0.022)	0.439
Contrast	0.026 (0.020–0.032)	0.027 (0.020–0.036)	0.592

All data are expressed as median (interquartile range). The *p*-values are the results of the Mann-Whitney test. AT, adipose tissue; GLCM, gray-level co-occurrence matrix; MTV, metabolic tumor volume; NGLDM, neighborhood gray-level difference matrix; SUV, standardized uptake value; TLG, total lesion glycolysis.

**Table 4:** ROC curve analysis of imaging parameters for predicting pathological complete response.

Parameter	AUC (95% Confidence Interval)	Cut-Off Value	Sensitivity (%)	Specificity (%)
Primary tumor				
Maximum SUV	0.580 (0.476–0.677)	10.77	58.3	59.5
MTV	0.561 (0.454–0.669)	3.48	30.6	84.7
TLG	0.534 (0.424–0.638)	85.90	38.9	73.0
Peritumoral AT				
First-order features				
Maximum SUV	0.561 (0.454–0.655)	2.45	61.1	56.8
Mean SUV	0.642 (0.531–0.731)	0.85	86.1	39.6
Standard deviation SUV	0.583 (0.469–0.677)	0.37	66.7	52.3
25th percentile SUV	0.633 (0.528–0.728)	0.45	52.8	74.8
50th percentile SUV	0.686 (0.583–0.768)	0.78	91.7	38.7
75th percentile SUV	0.645 (0.535–0.731)	1.04	86.1	41.4
SUV histogram kurtosis	0.586 (0.475–0.687)	7.04	25.0	93.7
SUV histogram skewness	0.600 (0.942–0.702)	1.35	50.0	69.4
SUV histogram energy	0.608 (0.501–0.706)	0.26	80.6	39.6
SUV histogram entropy	0.653 (0.552–0.750)	2.20	86.1	44.1
GLCM features				
Contrast	0.608 (0.486–0.707)	1.25	72.2	52.3
Correlation	0.505 (0.400–0.608)	0.64	97.2	12.6
Dissimilarity	0.622 (0.510–0.719)	0.73	72.2	52.3
Energy	0.607 (0.500–0.699)	0.11	86.1	36.0
Entropy	0.697 (0.585–0.791)	3.24	63.9	78.4
Homogeneity	0.717 (0.600–0.811)	0.75	58.3	85.6
NGLDM features				
Busyness	0.529 (0.419–0.630)	2.70	66.7	47.7
Coarseness	0.543 (0.434–0.643)	0.011	77.8	36.0
Contrast	0.530 (0.418–0.627)	0.023	41.7	69.4

AT, adipose tissue; AUC, area under the receiver operating characteristic curve; GLCM, gray-level co-occurrence matrix; MTV, metabolic tumor volume; NGLDM, neighborhood gray-level difference matrix; SUV, standardized uptake value; TLG, total lesion glycolysis.

**Table 5:** Univariate and multivariate logistic regression analyses of predictors for pathological complete response.

Parameter	Univariate Analysis		Multivariate Analysis	
	*p*-Value	Odds Ratio (95% Confidence Interval)	*p*-Value	Odds Ratio (95% Confidence Interval)
Primary tumor				
Maximum SUV	0.355			
MTV	0.380			
TLG	0.385			
Peritumoral AT				
First-order features				
Maximum SUV	0.258			
Mean SUV	0.016	23.70 (1.83–307.75)	0.012	26.92 (2.06–351.31)
Standard deviation SUV	0.174			
25th percentile SUV	0.025	29.49 (1.54–564.71)	0.139	
50th percentile SUV	0.004	55.27 (3.70–826.21)	0.002	76.37 (4.80–1215.22)
75th percentile SUV	0.015	12.47 (1.62–95.81)	0.015	12.46 (1.64–94.70)
SUV histogram kurtosis	0.074			
SUV histogram skewness	0.059			
SUV histogram energy	0.074			
SUV histogram entropy	0.029	3.98 (1.15–13.77)	0.034	4.11 (1.12–15.14)
GLCM features				
Contrast	0.426			
Correlation	0.840			
Dissimilarity	0.174			
Energy	0.037	0.02 (0.01–0.70)	0.083	
Entropy	0.001	3.49 (1.65–7.37)	0.001	3.67 (1.65–8.16)
Homogeneity	0.001	0.32 (0.16–0.63)	0.002	0.33 (0.16–0.66)
NGLDM features				
Busyness	0.402			
Coarseness	0.843			
Contrast	0.668			

AT, adipose tissue; GLCM, gray-level co-occurrence matrix; MTV, metabolic tumor volume; NGLDM, neighborhood gray-level difference matrix; SUV, standardized uptake value; TLG, total lesion glycolysis.

**Table 6:** Univariate and multivariate survival analyses for predicting PFS.

Parameter	Univariate Analysis		Multivariate Analysis	
	*p*-Value	Hazard Ratio (95% Confidence Interval)	*p*-Value	Hazard Ratio (95% Confidence Interval)
Primary tumor				
Maximum SUV	0.332			
MTV	0.002	1.01 (1.01–1.02)	0.039	1.01 (1.00–1.02)
TLG	0.008	1.00 (1.00–1.01)	0.178	
Peritumoral AT				
First-order features				
Maximum SUV	0.360			
Mean SUV	0.238			
Standard deviation SUV	0.600			
25th percentile SUV	0.310			
50th percentile SUV	0.022	8.60 (1.37–53.88)	0.083	
75th percentile SUV	0.224			
SUV histogram kurtosis	0.555			
SUV histogram skewness	0.535			
SUV histogram energy	0.221			
SUV histogram entropy	0.012	4.23 (1.38–12.93)	0.042	2.71 (1.10–10.54)
GLCM features				
Contrast	0.487			
Correlation	0.019	38.25 (1.83–796.39)	0.040	29.32 (1.17–734.56)
Dissimilarity	0.542			
Energy	0.418			
Entropy	0.045	1.87 (1.01–3.46)	0.437	
Homogeneity	0.546			
NGLDM features				
Busyness	0.998			
Coarseness	0.028	0.63 (0.42–0.95)	0.082	
Contrast	0.378			

All hazard ratios are expressed per 1.00 increase in parameter values, except for NGLDM coarseness. For the NGLDM coarseness, the hazard ratio values are expressed per 0.01 increase in the parameter values. AT, adipose tissue; GLCM, gray-level co-occurrence matrix; MTV, metabolic tumor volume; NGLDM, neighborhood gray-level difference matrix; SUV, standardized uptake value; TLG, total lesion glycolysis.

## Data Availability

The datasets generated during and/or analyzed during the current study are available from the corresponding authors upon reasonable request.

## Supplementary Materials

The following supporting information can be downloaded at: https://www.mdpi.com/article/10.3390/jpm14090952/s1, Table S1: Definition of textural features of peritumoral adipose tissue in the study.
