# Supplementary material for: Peritumoral Adipose Tissue Features Derived from [18F]fluoro-2-deoxy-2-d-glucose Positron Emission Tomography/Computed Tomography as Predictors for Response to Neoadjuvant Chemotherapy in Breast Cancer Patients
Source: J Pers Med. 2024 Sep 9;14(9):952. doi: 10.3390/jpm14090952 (PMC11432773; doi:10.3390/jpm14090952)
Supplement: Supplementary file 1 [file jpm-14-00952-s001.zip › jpm-3180328-supplementary.pdf]

Article title: Peritumoral adipose tissue features derived from FDG PET/CT as predictors for response to neoadjuvant chemotherapy in breast cancer patients

Authors: Jeong Won Lee, Yong Kyun Won, Hyein Ahn, Jong Eun Lee, Sun Wook Han, Sung Yong Kim, In Young Jo, Sang Mi Lee

Table S1. Definition of textural features of peritumoral adipose tissue in the study.

Textural features	Definition
First-order features	Describes the distribution of voxel intensities within the volume of interest through commonly used and basic metrics based on single-voxel analyses
SUVmax	Maximum SUV in the volume of interest
SUVmean	Average SUV in the volume of interest
SUVstd	Standard deviation SUV in the volume of interest
SUV Q1	25th percentile SUV in the volume of interest
SUV Q2	50th percentile SUV in the volume of interest
SUV Q3	75th percentile SUV in the volume of interest
SUV histogram kurtosis	Shape of the SUV distribution in the histogram relative to a normal distribution
SUV histogram skewness	Asymmetry of the SUV distribution in the histogram
SUV histogram energy	Uniformity of the SUV distribution in the histogram
SUV histogram entropy	Randomness of the SUV distribution in the histogram
GLCM features	Characterizes how often pairs of voxels with specific grey-level values found at a specific distance in a specific direction from 13 different directions
Contrast	Local intensity variation in the GLCM
Correlation	Linear dependency of grey-level values to their respective voxels in the GLCM
Dissimilarity	Variation of grey-level voxel pairs in the GLCM
Energy	Measure of homogenous patterns of grey-level voxel pairs in the GLCM
Entropy	Randomness of grey-level voxel pairs in the GLCM
Homogeneity	Homogeneity of grey-level voxel pairs in the GLCM
NGLDM features	Corresponds to the difference of grey-level between one voxel and its 26 neighbors in 3 directions
Busyness	Spatial frequency of changes in intensity
Coarseness	Level of spatial rate of change in intensity
Contrast	Spatial intensity change between neighboring regions

GLCM, grey-level co-occurrence matrix; NGLDM, neighborhood grey-level different matrix; SUV, standardized uptake value
